# Use of Artificial Intelligence in Imaging Dementia

**DOI:** 10.3390/cells13231965

**Published:** 2024-11-27

**Authors:** Manal Aljuhani, Azhaar Ashraf, Paul Edison

**Affiliations:** 1 Radiological Science and Medical Imaging Department, College of Applied Medical Sciences, Prince Sattam Bin Abdulaziz University, Al-Kharj 11942, Saudi Arabia; 2 Division of Neurology, Department of Brain Sciences, Faculty of Medicine, Imperial College London, London W12 0NN, UKpaul.edison@imperial.ac.uk (P.E.); 3 Division of Psychological Medicine and Clinical Neurosciences, School of Medicine, Cardiff University, Cardiff CF24 4HQ, Wales, UK

**Keywords:** Alzheimer’s disease, amyloid-related imaging abnormalities, artificial intelligence, dementia, neurodegenerative diseases

## Abstract

Alzheimer’s disease is the most common cause of dementia in the elderly population (aged 65 years and over), followed by vascular dementia, Lewy body dementia, and rare types of neurodegenerative diseases, including frontotemporal dementia. There is an unmet need to improve diagnosis and prognosis for patients with dementia, as cycles of misdiagnosis and diagnostic delays are challenging scenarios in neurodegenerative diseases. Neuroimaging is routinely used in clinical practice to support the diagnosis of neurodegenerative diseases. Clinical neuroimaging is amenable to errors owing to varying human judgement as the imaging data are complex and multidimensional. Artificial intelligence algorithms (machine learning and deep learning) enable automation of neuroimaging interpretation and may reduce potential bias and ameliorate clinical decision-making. Graph convolutional network-based frameworks implicitly provide multimodal sparse interpretability to support the detection of Alzheimer’s disease and its prodromal stage, mild cognitive impairment. In patients with amyloid-related imaging abnormalities, radiologists had significantly better detection performances with both ARIA-E (sensitivity higher in the assisted/deep learning method [87%] compared to unassisted [71%]) and for ARIA-H signs (sensitivity was higher in assisted [79%] compared to unassisted [69%]). A convolutional neural network method was developed, and external validation predicted final clinical diagnoses of Alzheimer’s disease, dementia with Lewy bodies, mild cognitive impairment due to Alzheimer’s disease, or cognitively normal with FDG-PET. The translation of artificial intelligence to clinical practice is plagued with technical, disease-related, and institutional challenges. The implementation of artificial intelligence methods in clinical practice has the potential to transform the diagnostic and treatment landscape and improve patient health and outcomes.

## 1. Introduction

Dementia is an umbrella term used to describe a range of symptoms occurring, including deterioration in memory and other cognitive domains. These are considered sufficient to significantly impair the activities of daily living memory [1]. Dementia is most likely caused by a combination of age-related alterations, genetics, environment, and lifestyle choices. It is estimated that the global number of people suffering from dementia will rise from ~57 million cases in 2019 to ~153 million cases by the year 2050. Even though the number of individuals living with dementia was projected to increase substantially, age-standardized prevalence for both sexes was stable between 2019 and 2050. In 2019, it was estimated that there were more women with dementia than men with dementia worldwide (the female-to-male ratio was almost double), and this pattern is expected to persist until 2050. The new cases projected are heterogeneously distributed geographically, with the smallest percentage increases in high-income Asia Pacific (53%) and Western Europe (74%) and largest in North Africa and the Middle East (367%) and Eastern sub-Saharan Africa (357%). Increases in cases are projected to be driven predominantly by population growth and population aging, but the relative contribution of the two was different in each world region, with population growth the main driver of increases in sub-Saharan Africa and population aging driving most of the increases in East Asia [2]. Alzheimer’s disease (AD) is the most common cause of dementia in the elderly population (aged 65 years and over), followed by vascular dementia, Lewy body dementia, and rare types of neurodegenerative diseases, including frontotemporal dementia (FTD) [3,4,5,6]. There is an unmet need to improve diagnosis and prognosis for patients with dementia, as cycles of misdiagnosis and diagnostic delays are challenging scenarios in neurodegenerative diseases. This has a domino effect where diagnostic errors lead to incorrect treatments being prescribed, while delays in receiving diagnoses prevent identification of patients eligible to partake in clinical trials. Geographical variability in receiving timely diagnosis and adequate management of neurodegenerative diseases stems from the lack of disease awareness among healthcare professionals, patients, and their care partners. Moreover, limited availability of resources and specialists, underfunding, and stigma around diagnoses further delay a timely diagnosis [7]. With the advent of disease-modifying treatments, landscape changes in the research and medical arenas are required. The goal is to ensure timely and accurate diagnosis, which is essential to guide treatment selection and increase the participation of eligible patients for clinical trials. Since neurodegenerative diseases are multifactorial and complex in nature, with significant individual heterogeneity in the underlying genetics and biology, a precision medicine (e.g., pharmacogenomics) approach is warranted. This will allow patient stratification to enable patients to receive timely treatment that will likely be beneficial while avoiding treatment of individuals at risk of experiencing severe side effects (who can be administered alternative treatments).

Neuroimaging is routinely used in clinical practice to support the diagnosis of neurodegenerative diseases [1]. Magnetic resonance imaging (MRI) is used to examine the brain structure, longitudinal patterns of atrophy, and alterations in brain function. Positron emission tomography (PET) measures metabolic activity using radioactively labelled ligands. Computed tomography (CT) is a computerized X-ray imaging procedure that generates cross-sectional images or “slices”. Electroencephalogram (EEG) records the changes in electrical activity of the brain over time. Clinical neuroimaging is amenable to errors owing to varying human judgement, as the imaging data are complex and multidimensional. Although visual rating scales, including medial temporal lobe atrophy and white matter hyperintensity load, have been informative in interpreting clinical neuroimaging, the enriched data processes yielding multiplex data suggest that multiple informative features may not be detectable through human clinical observation or measurement. For instance, resting-state functional magnetic resonance imaging (fMRI) can derive a plethora of connectivity metrics between thousands of nodes, which are amenable to machine learning approaches. Deep learning methodologies have demonstrated superiority in interpreting clinical data dimensions compared with human neuroimaging interpretation [8].

Artificial intelligence algorithms enable automation of neuroimaging interpretation and may reduce potential bias and ameliorate clinical decision-making. A form of machine learning, deep learning, is well adapted to handle the neuroimaging analyses, owing to its high dimensionality, non-linear nature, and high covariance within the data. While several studies have assessed the potential of machine learning processes in deciphering neuroimaging features indicative of cognitive diagnoses or conversion to dementia [9], there is an unbridged gap between the research and clinical application of artificial intelligence. A cloud of uncertainty remains regarding the use of machine learning processes to best inform clinical decision-making in juxtaposition to human decision-making.

The aim of this review is to understand the potential applications of artificial intelligence in extracting neuroimaging features in neurodegenerative diseases, their methodological constraints, and highlight future directions to facilitate clinical implementation of artificial intelligence methods. Artificial intelligence holds promise to revolutionize the arcane field of neurodegenerative diseases through timely diagnosis, monitoring, and treatment of neurodegenerative diseases.

## 2. Methodology

PubMed and Google Scholar were used as the main search engines. The key search terms included “artificial intelligence”, “machine learning”, “deep learning”, “neural networks”, “neuroimaging”, “dementia”, “Alzheimer’s disease”, “dementia with Lewy bodies”, and “Parkinson’s disease”, “amyloid-related imaging abnormalities”. The purpose of the research was to evaluate artificial intelligence approaches used in dementia; detection of amyloid-related imaging abnormalities as part of safety monitoring of patients on anti-amyloid treatment; differential diagnosis; challenges; and future opportunities with implementing artificial intelligence approaches in the clinic.

The novel aspect of this review is that it discusses how artificial intelligence approaches may be utilized in the real world to monitor treatment efficacy and safety of anti-amyloid treatments and includes a section on differentiating between dementias, particularly dementia with Lewy bodies, from Alzheimer’s disease and Parkinson’s disease dementia. The review then emphasizes the (novel) strategies that can be adopted to implement artificial intelligence approaches in the clinic.

## 3. Artificial Intelligence Methods Used in Dementia

**Figure 1:**
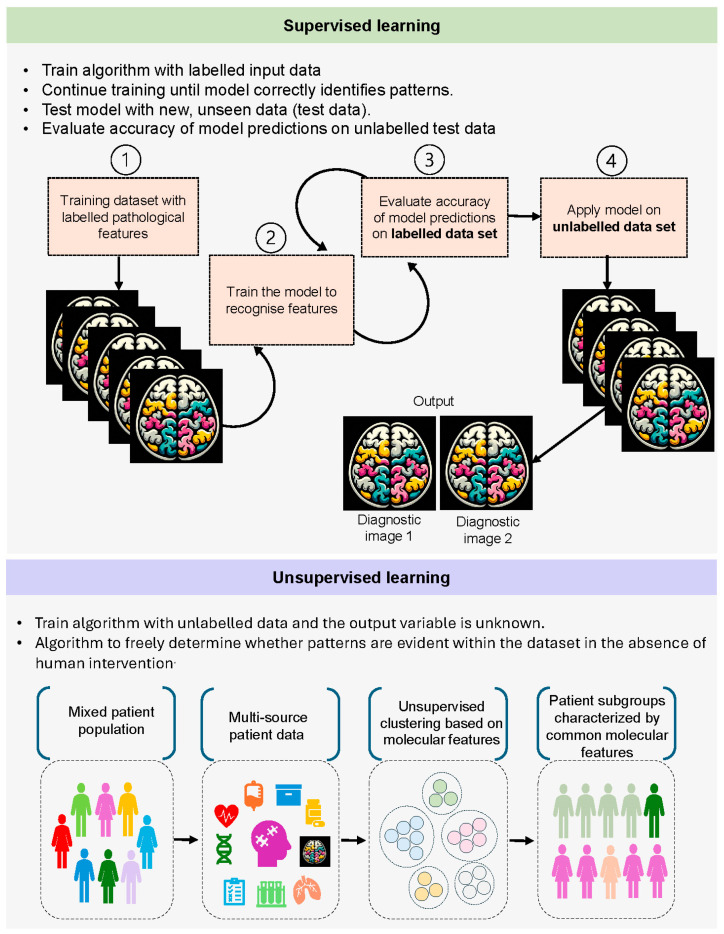
The common learning processes involve supervised and unsupervised learning.

The rationale of using artificial intelligence is to develop computer algorithms to emulate human functioning. Two subsets widely used in neurodegenerative diseases include machine learning and deep learning [9]. Machine learning utilizes algorithms that can recognize key data patterns and translates that knowledge to predict novel information. Common learning processes include supervised and unsupervised learning (Figure 1). Supervised learning is where the algorithm uses input data and corresponding output labels with which it fine-tunes itself till the model becomes efficient to establish valid patterns between two sets of datasets (training features) [10]. These test data are used to evaluate the trained model with new data that it has not seen before, and this unlabeled test data are used to make predictions about future instances. Unsupervised learning trains a model on unlabeled input data, where the correct output variable is not known. This liberates the algorithm to decide for itself if there are patterns in the datasets, free of human intervention [10].

Deep learning is a complicated asset of machine learning, which involves a convolutional neural network architecture for logical data analyses akin to the way the human brain functions. The main differentiating factor between deep learning and machine learning is that the former utilizes neural networks, which require minimal human intervention and are equipped to handle the larger data requirements. Deep learning processes raw input data directly, while machine learning usually requires raw data to be pre-processed. The rationale for the development of artificial intelligence methods (machine learning and deep learning) is to create algorithms that identify, analyze, and extract data from neuroimaging to detect neurodegenerative diseases with a high specificity and sensitivity [9].

## 4. Timely Diagnosis of Alzheimer’s Disease

**Figure 2:**
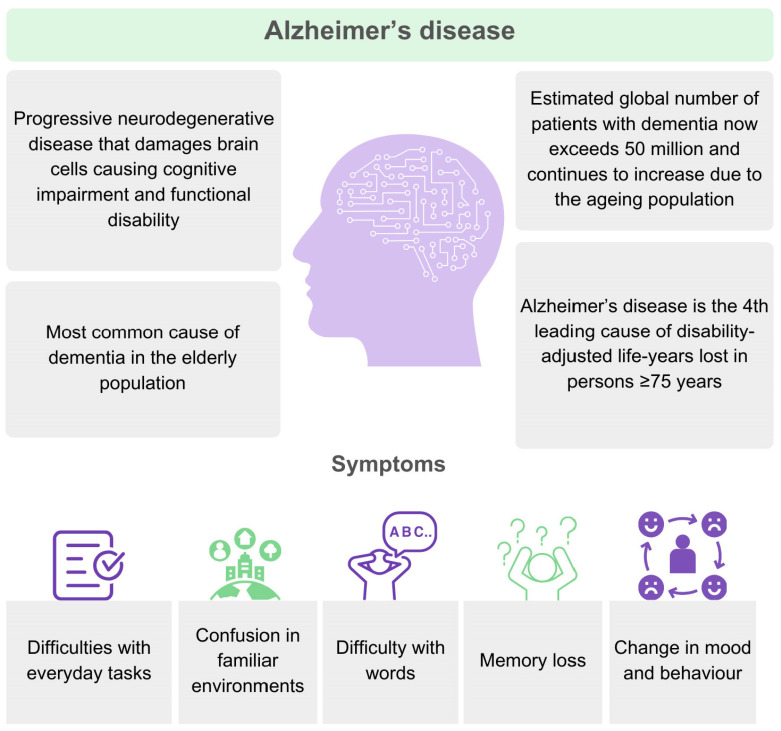
Alzheimer’s disease is the most cause of dementia. It is a long-term and progressive disease affecting ability to learn, think, remember, and reason.

On 3 November 1906, a clinical psychiatrist and neuroanatomist, Alois Alzheimer, reported “A peculiar severe disease process of the cerebral cortex” to the 37th Meeting of South-West German Psychiatrists in Tubingen. He reported on a 50-year-old woman whom he had followed from hospital admission for paranoia, progressive sleep and memory disturbance resulting in aggression and confusion up to her death five years later. In his report, the brain histology was characterized by specific Aβ plaques and neurofibrillary tangles [11]. A disorder of the central nervous system, AD is a chronic syndrome characterized by progressive loss or reduction in two (or more) cognitive domains (Figure 2). It leads to gradual losses in cognitive ability, including decision-making, language, memory, and learning orientation, affecting judgement [12]. Patients with AD experience cognitive impairment from mild to severe [13]. The earliest presentations may be subjective cognitive decline at the time of normal performance on objective measures [14]. Mild cognitive impairment (MCI) is defined as the symptomatic predementia stage on the continuum between normal cognitive aging and dementia that initially affects primarily memory, but it can also involve other cognitive domains [15]. In contrast, dementia is characterized by cognitive impairment that interferes with independence and daily activities. Dementia with a typical clinical phenotype of progressive disease with early and prominent amnestic features is conceptualized as prototypical for Alzheimer’s disease (AD) [1].

**Figure 3:**
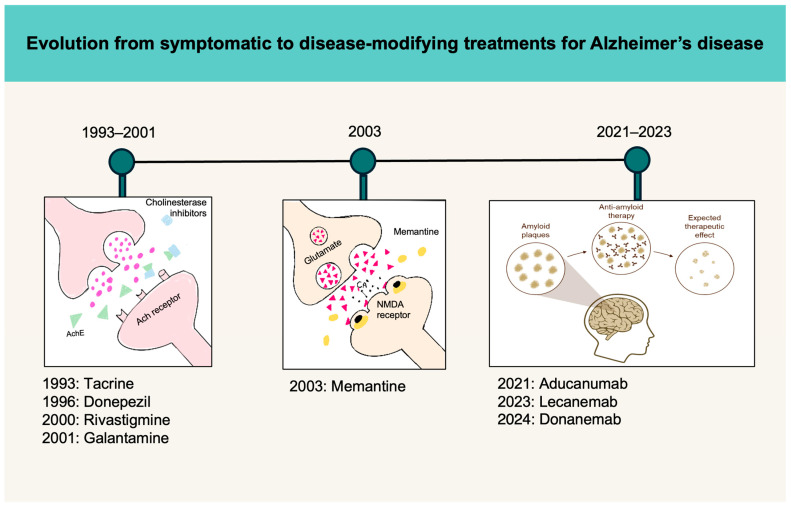
The early pharmacotherapies were aimed at symptomatic relief in patients with Alzheimer’s disease. Recently, the field has made a paradigm shift with the advent of disease-modifying treatments targeting β-amyloid.

The amyloid cascade hypothesis (1992) remains the most widely accepted hypothesis in AD. It posits a serial model of causality where Aβ initiates a cascade of events leading to tau hyperphosphorylation, cellular atrophy, and vascular events, which manifests in the form of clinical dementia [16]. The field of AD has seen a paradigm shift from emphasis on symptomatic treatments to disease-modifying therapies (Figure 3). Two anti-Aβ therapies—donanemab (Eli Lilly & Co., manufactured in Limerick, Ireland; approved in USA) and lecanemab (Eisai Co., Ltd. [Tokyo, Japan] and Biogen Inc. [Cambridge, MA, USA]; approved in USA, Japan, China, and UK)—have received standard approval by the FDA for the treatment of early AD (aka MCI and mild AD) [17,18,19]. The onus has shifted towards trialing therapies in early AD, as the therapeutic window of opportunity may still be present, while in moderate and severe cases, this window of opportunity may be lost as evident from the lack of efficacy in previous anti-Aβ trials.

## 5. Artificial Intelligence Approaches in Alzheimer’s Disease

With the global availability of disease-modifying therapies on the horizon and an expanding drug pipeline, timely diagnoses that enable timely treatment is the need of the hour [20,21]. Neuroimaging is an essential tool for identifying biomarkers of AD for early diagnosis and intervention. As neuroimaging rapidly advances, a challenge in analyzing and interpreting diverse imaging data presents itself. Computer-aided algorithms for integrative analyses through artificial intelligence will be required to overcome these limitations. Artificial intelligence integrates complex multimodal data, improving the accuracy of biomarker testing and potentially providing accurate and widely accessible, timely diagnosis of AD [9,22]. Multiple studies have developed artificial intelligence-based algorithms for grouping, examining, and diagnosing AD and identified non-invasive, early potential AD biomarkers (Table 1) [9,22]. Machine learning algorithms classified AD upon detecting abnormal hippocampal functional connectivity, yielding an accuracy of more than 80% to discriminate between cognitively normal (CN), MCI, and AD [23]. A densely connected convolutional neural network with a connection-wise attenuation mechanism predicted a diagnosis of AD with a high accuracy on MRI scans [24]. Machine learning algorithms enabled differentiation of AD from other neurodegenerative diseases such as FTD and vascular dementia [25,26]. A multimodal deep neural network using structural magnetic resonance imaging and fluorodeoxyglucose-PET (FDG-PET) images was able to classify patients with a clinical diagnosis of probable AD and non-demented CN and identify patients with MCI who converted to AD within 1–3 years [27]. Simple and fast machine learning models, such as using a histogram as the feature extractor and random forest as the classifier, yielded high accuracy for the detection of automatic AD [9]. A deep learning-based method with an fMRI dataset predicts early and late MCI, as well as AD [28].

Artificial intelligence can generate models from multiple AD biomarkers to enable a timely and a high-accuracy diagnosis. Analyses of biomarkers from brain MRI and single nucleotide polymorphism (SNP)-based genetic data demonstrated that the genetic data were superior in detecting progression of AD compared with MRI [30,31]. The MRI data suggested altered brain anatomy to better categorize patients with MCI compared with the genetic data. Combinatorial use of genetic and imaging data improved prediction of AD progression [31]. Since cognitive deterioration is associated with higher Aβ-PET, hippocampal volumetric reduction on MRI, glucose hypometabolism on FDG-PET, and increased tau levels [32,33], a study combined the multimodal data to develop a biomarker-based machine learning algorithm to predict progression of cognitive deterioration in AD [34]. A support vector regression model was trained with Aβ-PET, structural MRI, and cerebrospinal fluid data from 121 individuals with autosomal-dominant AD for predicting the estimated years to symptom onset, a proxy of cognitive decline. Autosomal-dominant AD offers a distinctive training dataset as the disease onset is at relatively early stages, which makes it less likely for age-related morbidities, for example, small vessel disease and transactive response DNA binding protein of 43 kDa (TDP-43), to confound the development of AD pathology and cognitive decline. Since the time to develop dementia symptoms is genetically driven, estimated years to symptom onset serves as a proxy for AD-related cognitive decline. The trained model was tested on 216 individuals with sporadic AD, where a high prediction accuracy for 1–4 year global cognitive and memory deterioration was demonstrated. The model may also significantly reduce sample size requirements in clinical trials. Future studies should include tau-PET and neuroinflammatory biomarkers to improve predictive accuracy of potential cognitive decline [34].

A novel method, DANMLP, which is a combination of a dual attention convolutional neural network and a multiplayer perceptron for the diagnosis of AD, has been proposed for the computer-aided diagnostic process by aggregating multimodal data from structural MRI, clinical information containing demographics and neuropsychology test scores, plus apolipoprotein E (APOE) genotype. In particular, the DANMLP introduces key components: patch-convolutional neural networks to learn image features from local patches; position self-channel and channel self-attention for capturing inter-patch feature dependency in pixel space attention and position awareness mechanism; finally, two multilayer perceptron networks to derive clinical information and output data used to classify AD. Compared with other state-of-the-art methods, DANMLP achieved accurate classification. Delineated focal visualization of regions could lead to early AD diagnosis by clinicians. This implies that DANMLP can be used effectively to diagnose individuals with both AD and MCI [35]. Further studies will need to focus on the categories of AD-related diagnosis by including subjective cognitive decline, early and late MCI. In addition, since genes play a central role in the development of AD and there are abundant gene expression data available, it would be helpful to design a method that could combine neuroimaging with gene expression for studying correlations between different brain regions and various sets of target genes associated with AD pathogenesis.

A deep learning algorithm was applied to the FDG-PET brain images of 1002 patients (acquired 2005–2017), which predicted a clinical diagnosis of AD in an average of 75.8 months preceding the final diagnosis, with 82% specificity at 100% sensitivity [36]. A deep learning algorithm may predict AD early in the disease process, complemented by molecular biology and neuroimaging tests, maximizing the chances for a timely therapeutic modulation. The advantage of a deep learning algorithm is that it utilizes the whole brain to extract various information from different brain compartments to reach its ultimate decision. The algorithm classifies pixel-by-pixel volumes of the brain into diagnoses, which may ensure consistency and eliminate the subjective human interpretation, potentially minimizing cycles of misdiagnosis. The algorithm’s performance on the general patient population remains to be proven, as the existing study was exploratory. Future studies should test the performance and robustness of the algorithm in prospective, unbiased, real-life scenario patient cohorts. Additional validation with larger and prospective external test sets should be performed prior to clinical recommendation. Tailor-made 3D convolutional neural networks accurately classified FDG-PET brain scans between AD, FTD, or CN individuals. The model outperformed clinical interpretation by the experienced physicians, which may be a useful addition to clinical practice once validated [29].

Individuals with AD display distinct brain imaging phenotypes, which may signal disease subtypes detectable using MRI and machine learning methodologies. There is a lack of biological interpretability if these subtypes are not driven by genes or elements conferring susceptibility. To dissect disease heterogeneity through phenotypic and genetic data, a multi-view deep clustering model with only weak supervision (Gene-SGAN) was developed, capturing the genetically distinct subtypes of each trait along with endophenotype signatures. The patient-derived phenotypes differed significantly in neuroanatomical patterns, genetic determinants, and biological and clinical markers, indicating different underlying neuropathological processes and drivers of the intrinsic genes, as well as most susceptibility factors [37]. Four subtypes of AD were deduced, each with unique genetic associations and different features in alternative clinical phenotypes as well as cognitive performance; key demographic variables included levels of Aβ and phosphorylated-tau (Table 2). The potential clinical impact of combining neuroimaging and SNP data is multifaceted. It enables dissection of disease heterogeneity into neuroanatomical subtypes that are genetically mediated and functional, facilitating precise diagnosis with significant downstream effects. For instance, the capabilities of various levels and scales to classify disease could be enhanced by identifying biologically robust subtypes associated with a particular disease. This simulation approach provides novel methods of classifying patients and assessing the efficacy in future clinical trials, particularly given that anti-Aβ treatments have shown inconsistent results and are limited by their capacity to reach other cellular compartments. These artificial intelligence methods may provide a valid endophenotype using which processes of drug repurposing and delivery will become easier. The MCI and AD subtypes used by Yang et al. (2024) were taken only from a moderate cohort of 1061 individuals [37]. Given that multi-center large-scale imaging-genomic consortia of AD, as exemplified by AI4AD (http://ai4ad.org/), can likely consolidate related disease subtypes with improved reproducibility and greater diversity in genetic-structure patterns, it is plausible to expect the generation of additional SNPs. SNPs may be incorporated based on the information from genes that are amenable to therapeutic modulation, providing detailed insights into drug discoveries.

The interconnection of brain regions in neurological disease contains key information for the development of biomarkers and diagnostics. Graph convolutional networks (GCNs) are common for discovering functional brain connection patterns indicative of disease states. GCNs were developed for two different purposes by Han et al. (2024), who presented a resting-state fMRI-based binary classifier using a functional connectivity-based GCN framework: (1) MCI diagnosis: used to classify MCI from CN; and (2) dementia risk prediction: used to classify CN from at-risk subjects that have the potential for developing MCI but are not clinically diagnosed as having MCI [38]. The experiments yield several important findings: the proposed GCN produces better performances than both the baseline GCN and the support vector machine (SVM). It not only obtained the highest average accuracy of 80.3% (11.7% higher than the baseline GCN and 23.5% higher than SVM) but also achieved the best result with an accuracy of 91.2%. Secondly, the overall performance of the GCN framework with (absolute) individual functional connectivity was slightly better than that with global functional connectivity. Nevertheless, global graphs with appropriate connectivity deliver at least the same or even better performance than individual graphs using GCN in some scenarios, which establishes that performance heavily relies on suitable richness of connectivity. Moreover, evidence suggests that the strength of self-network connection in some regions of brain networks (e.g., default mode network, ventral attention network, visual network, and somatomotor network) would probably be related more with GCN classification.

In a cross-sectional study, resting-state fMRI, clinical assessments, and neuropsychometric evaluations were performed to detect dementia subtypes with distinct functional connectivity associated with neuropsychiatric subsyndromes [39]. Sparse canonical correlation analysis led to the identification of two robust subsyndromes, behavioral and anxiety, in neuropsychiatric symptoms-functional connectivity-correlated latent space with distinct connectivity patterns and characteristic phenotypes, including disability in daily living. Using these neuropsychiatric symptoms, subsyndrome–associated functional connectivity latent features subsequently clustered participants into three subtypes and observed substantial differences in clinical assessments, baseline connectivity patterns, and longitudinal clinical progression across dementia subtypes. Significantly, patients in subtypes 1 and 2 had worse cognitive deficits at baseline and greater decline in cognition over time relative to controls, whereas patients with subtype 3 showed brain and cognitive phenotypes like healthy individuals from the control group. These data highlight the potential of targeted interventions that address specific cognitive impairment and dysfunction present across dementia subtypes.

The promise of imaging-derived multimodal connection patterns remains mostly untapped. Zhou et al. (2024) present a GCN-based framework (SGCN) that implicitly provides multimodal sparse interpretability to support the detection of AD and its prodromal stage, MCI [40]. The sparse regional importance probability learned by SGCN was able to identify signature regions of interest (ROIs) while the connective importance probability uncovered disease-specific brain network connections. SGCN was evaluated on the ADNI database with multimodal brain images (VBM-MRI, FDG-PET, and AV45-PET), and the ROI features encoded by SGCN were shown to be improved for AD status identification. The detected abnormalities are strongly associated with clinical AD features (MMSE, ADAS-Cog-13, CDR-SOB). The identified brain dysfunctions in terms of large-scale neural systems and sex-related connectivity were interpreted as abnormalities in AD/MCI. The prominent brain connectivity abnormalities and the salient ROIs interpreted by SGCN appear to be significant for forming new biomarkers. These observations improve multimodal diagnosis in the network-based disorder and allow for precision diagnostic possibilities. SGCN introduces saliency ROIs to identify subject conditions between CN, AD, and MCI and provide an ordering of the relative prominence of subgraph structure for discriminating subjects, which achieved superior prediction performance. Second, SGCN interprets brain regions and brain connectivity using the importance probability method, which is further verified by large-step statistical analysis on the topological patterns learnt. However, potential limitations of these methods are their robustness and generalizability to other neurodegenerative disease datasets. Diverse atlases could substantially affect the classification of disorders such as AD and MCI based on ROI-based analysis. Further studies are required to explore the extent to which these biomarker findings generalize across brain atlases. Further application of the SGCN model is warranted to enhance prediction on the conversion time and whether and when the MCI will be converted into AD based on longitudinal data for more clinically relevant needs targeted towards the derivation of AD [40,41].

As mentioned previously, advancing early identification of dementia, including AD, is a healthcare priority. Ávila-Jiménez et al. (2024) leveraged deep learning models (by developing an artificial neural network) and comprehensive medical records to discern subtle disease patterns, which facilitated early detection [42]. These models have the potential to optimize research allocation, enhance diagnostic accuracy, and the quality of care. The application of deep learning in medical diagnostics may positively transform patient outcomes and ensure efficiency in the healthcare system.

Comparative studies involving machine learning approaches in dementia are warranted. Briefly, logistic regression is a method that has been widely used in traditional statistics for predicting binary outcomes based on one or more predictor variables [43]. Logistic regression has been adopted as well by machine learning practitioners, where logistic regression is commonly used to classify new data points based on training data. Random forest is a machine learning technique that extends the concept of decision trees by putting together in a single model an ensemble (or forest) of decision trees that cooperate with each other. The model generates predictions from each decision tree, and the random forest algorithm takes the result of the maximum trees as a result. The goal of the algorithm is to create several decision trees that are as uncorrelated as possible and/or can make predictions more accurately than any decision tree alone. The number of trees in the random forest can be varied, and how complex trees are allowed to become determines the optimal combination that works best. When a random forest algorithm is used, other useful data can be mined using scikit-learn, including feature importance showing which variables in the model were most important for the model’s predictive power. Higher value means higher priority. A neural network is a machine learning algorithm that models itself after the communication between neurons in the brain so it can recognize patterns and relationships in datasets. The algorithm works on the concept of multiple layers: input layers, output layers, and hidden layers in between. Less the hidden layer and number of nodes of any layer can be modified. A node acts similarly to a neuron when the network is trained, as it will be either turned on (activated) or off (inactive). Several hidden layers are tried with multiple nodes to see what gives the best prediction. A study involving 1491 older adults evaluated three popular machine-learning algorithms—logistic regression, random forest, and neural network algorithms—on their ability to estimate future dementia cases from a handful of known factors for dementia and cognitive decline [43]. Results indicated random forest outperforms logistic regression and neural networks in most of the performance metrics. Random forest has the highest number of true positive predictions and the lowest number of false negative predictions among all assessed algorithms. In contrast, neural networks produced the most true negatives and the least false positives. On balanced accuracy, precision, recall, F1 score, and Matthews correlation coefficient, random forest was the best classifier with higher scores than logistic regression and neural networks. On the other hand, logistic regression had a greater area under the curve than random forest and neural networks. Similarly, The Sydney memory and ageing study with 1037 individuals deemed random forest algorithms as ideally placed to handle data on predictors of dementia onset [44]. Machine learning methods (repeated linear regressions, penalized linear regressions, random forests, and neural networks) were compared in a Health and Retirement Study to determine the social determinants of health [45]. Marginal performance gain by using more complex machine learning models to fit the data was observed compared with simpler models. Neural networks, on the other hand, were far superior to the three other methods and predicted the data well. Automated feature engineering techniques improve prediction and/or fit over simpler models to varying extents, but some of the machine learning methods do not improve anything beyond these simpler models. The predictors that emerge across the models provide insight into potential social determinants of health that are saliently predictive of biological indicators for chronic disease and imply that the non-linear and interactive relationships between variables inherent to the neural network approach may be important features.

Taken together, neural networks and random forests provide two insights that could be useful in dementia research [43]. Essentially, neural networks are indispensable to imaging-based diagnostics, whereas random forests offer generalization and interpretability for structured data. A combination of both could act as a more holistic artificial intelligence-driven diagnostic for dementia. Neural networks are complicated and labeled as “black boxes” as they learn features on their own but show little to no transparency behind the reasoning of their answer. The problem is that in clinical settings, physicians want explainable insights, and this complication becomes a limitation. Some early techniques, such as saliency maps or Grad-CAM, were a first step to gain trust by doctors in decisions made by artificial intelligence, allowing partial decoding of the neural network decision process view [46]. Random forests are generally more interpretable and can indicate the most important features (e.g., cognitive assessments, genetic markers) within the data that contribute to predicting outcomes related to dementia. The feature transparency of this characteristic makes it easier for clinicians/researchers to understand the information or attributes on which the model predictions are based, thus it may facilitate research and individualized patient care. This makes random forest models especially attractive for early-stage screening and risk assessment. Although neural networks perform well in complex image analysis, they depend upon a large neuroimaging dataset to generalize properly and avoid overfitting. In neuroimaging, a limitation is the scarcity of data because there are limited high-quality datasets for dementia. In dementia research, available datasets are small, so neural networks often use data augmentation or transfer learning (pre-trained models) to mitigate these problems. Random forests are preferable for smaller datasets, and overfitting is less likely in practice. They are better suited than many other methods for datasets combining neuropsychological tests and demographic data, as they do not need extensive amounts of data for training, whereas sample sizes in these types of studies are often smaller than those available at imaging centers. Neural networks are known for their high accuracy in several neuroimaging tasks, especially if there is enough training data that can help the model distinguish between different dementia subtypes and stages. However, this may limit their generalization to datasets collected in diverse imaging centers since variability in scanning protocols and equipment is known to exist. Random forests are a model deployment that is not specific to any one data source while remaining resistant to overfitting on data, finding them well validated across dementia research in terms of generalizability capabilities. But for more subtle image classification tasks, they might have lesser performance because they do not utilize neural networks hierarchical feature extraction ability [43,47].

Electronic health records are a rich source of longitudinal data that can decipher patterns in arcane diseases like AD. Data present in clinical records represent a clinician’s understanding and interpretation of their patient’s clinical history pre-diagnosis or imaging, which enables cost-effective implementation of a model to stratify a patient’s risk in primary care. With significant advances in informatics and curated multi-omics data, integrative approaches are warranted to acquire valuable disease insights. Diverse biological knowledge networks prioritize the translation of biological hypotheses through synthesized knowledge across different modalities into shared clinical associations of significance. Knowledge networks applied to electronic health records from the University of California, San Francisco, predicted AD onset, prioritized biological hypotheses, and contextualized sex dimorphism [48]. Random forest models were trained, which led to the identification of 749 patients with AD and 250,545 individuals who were CN with a 72% predictive power up to 7 years prior to the disease onset. Adopting a precision medicine approach, the Scalable Precision Medicine-Oriented Knowledge Engine was used to identify diseases with predictive power preceding AD onset. Knowledge networks highlighted mutual genes between multiple top predictors and AD (e.g., APOE, interleukin-6, β-actin, and insulin). Genetic colocalization analyses supported the association of AD with hyperlipidemia at the APOE locus and a female AD correlation with osteoporosis at a locus in the proximity of membrane-spanning 4-domain A6A. This is an example of how clinical data can predict AD early and can be used to derive personalized biological hypotheses [48]. There are a few challenges of using electronic health records coupled with artificial intelligence approaches. Electronic health record data complexity and quality may impact prediction models, and it is difficult to dissect how the involvement of health care professionals and patients’ attitudes and behaviors, societal factors, and/or underlying biology affect feature delineation. Matching may aid interpretation through the removal of the non-biological covariates, though longitudinal validation of hypotheses via different omics data types will be required. Due to the dynamic nature of patient demographics, societal and biological factors, continuous training of prediction models will be required along with regular updates and evaluation if adopted in the clinic. This will ensure effective utilization and factor in biases emerging from the learned data. Model utilization should consider the effect of cohort selection biases and matching methods on the model’s generalizability. Retraining and calibration need to be a routine part of model application for it to adaptively handle the hypothetical data drifts as well as fast-changing future clinical practice strategies. The data inside a clinical electronic health record may be old and/or limited. This could offer a one-off surface interval picture of someone’s health; caution must be made in that not having a record does not equate to the absence of condition, and historical medical data may no longer be available in the electronic health record. An electronic health record is a snapshot of part of someone’s medical history and would be expected to reveal chronic or common diseases, as well as their typical treatments and lab measurements. Further work needs to explore the influence of changes in data representation that can accommodate for sparsity in data, continuous outcomes of laboratory results, and competent temporal assignment at onset time. Clinical diagnosis goes beyond binary representation or relies on drug prescriptions with descriptions for diagnostic assignments. Future work should aim to address the heterogeneous nature of neurodegenerative diseases, including AD, through identifying subgroup-specific features, with divisions based on biotype, dementia syndromes, racialization, etc. Applications using hierarchical models might make it easier to transfer learning or fine-tuning on a subpopulation, thus increasing the personalization of models. This area needs research to determine propensity for diagnostic-relevant AD-associated comorbidities, or those with the potential to begin after progression of an AD process. Work is needed to study potential causality using Mendelian randomization or mechanistic studies.

## 6. Monitoring Treatment Safety in Alzheimer’s Disease

Monoclonal antibodies, including lecanemab, directed against aggregated forms of Aβ can cause amyloid-related imaging abnormalities (termed ARIA) detectable on an MRI scan [49]. Two types of ARIAs characterized as ARIA with oedema (ARIA-E) and ARIA with hemosiderin deposition (ARIA-H) are reported. The time and occurrence of ARIA are treatment dependent. The majority of reported ARIA cases are asymptomatic, but symptomatic cases are also possible. Although rare, severe events, including fatal events may occur [50]. According to Alzforum news (www.alzforum.org) dated 26 January 2024, an estimated 2000–3000 patients across the United States are on lecanemab. Accurate ARIA detection is required to monitor and make informed treatment choices to inform monitoring and treatment decisions, e.g., dose interruptions [51].

ARIA detection and radiographic severity assessments remain a challenge. Analysis of trials has shown that 31% of ARIA-E events were not identified by local radiologists and 42% were not found at central reading in original clinical programs [52,53]. A different investigation described that the miss rate by on-site radiologists of ARIA cases was 84%, for which all but one were noted after rereading (14%) [54]. Radiologists with less experience of ARIA are less likely to recognize the majority of cases. A new deep learning software correctly identified ARIA-E and ARIA-H and radiographic severity [55]. To prove the clinical utility of the assistive reading and interpretation system, a diagnostic study was also performed in a multiple-reader–multiple-case design. Radiologists had significantly better detection performances with both ARIA-E (sensitivity higher in assisted [87%] compared to unassisted [71%]) and for ARIA-H signs (sensitivity was higher in assisted [79%] compared to unassisted [69%]). This was true with respect to mild cases (ARIA-E sensitivity: assisted, 70%, vs. unassisted, 47%), for which imaging findings can be relatively subtle [55]. The assistive software tool was related to increased diagnostic accuracy in the identification of ARIA, compared with unassisted radiographic evaluation for both general and specialized radiologists. This can provide potential means by which reliable monitoring of ARIA might be achieved more widely within clinical practice. Assistive software is not intended for the diagnosis of neuroimaging and should only be used by qualified radiologists. Knowledge of the potential for ARIA and training in use with certain assistive device software tools can help create greater awareness, translation (via automated speech recognition systems), and adoption practice patterns to provide more sensitive detection of ARIA, thereby augmenting safe management practices within treatment settings when patients are receiving anti-Aβ-directed antibody therapies.

## 7. Differentiating Dementia with Lewy Bodies (DLBs) from AD and Parkinson’s Disease with Dementia (PDD)

DLB is prevalent in approximately 25% of dementia cases [56]. DLBs have characteristic symptoms, such as visual hallucinations, Parkinsonism, and rapid eye movement-sleep behavior disorder. Individuals with DLB are three times more frequently affected by falls than those with AD [57]. An accurate diagnosis is challenging, particularly early in the disease process, owing to heterogeneous clinical presentation [58]. Owing to the common co-occurrence of psychosis and Parkinsonism, DLB may be mis- or under-diagnosed. DLB is markedly under-diagnosed and frequently mistaken for AD until pathological confirmation on postmortem examination [59]. Clinically, the course and prognosis of DLB is more aggressive than AD. For example, the estimated survival time in DLB patients is significantly shorter than in AD patients and can be as short as two years [60]. A review of the electronic health records in London for 500 patients meeting criteria for a DLB diagnosis identified that only half received this diagnosis. Only five people had imaging, which is now recommended to support diagnosis [58,61]. There is striking divergence between the United Kingdom and their European counterparts. European collaborative studies show around 60–70% of individuals are imaged through the diagnostic work-up [61]. Patients with DLB treated with antipsychotic drugs have a higher risk of death and worsening Parkinsonian symptoms. The treatment of DLB is further complicated by heterogeneous responses in individuals to medications commonly used for AD and PD [61].

Core diagnostic features of DLB are common in PDD. PDD is diagnosed using consensus report criteria of the Lewy Body Consortium (≥1 year between Parkinsonian motor symptoms and dementia diagnosis) [58]. Neuropathologically, biomarker and imaging studies have shown AD-type pathology to be a stronger determinant of DLB than PDD [62]. On the converse, most cases of PDD demonstrate mild or no AD pathology. Synucleinopathy per se is probably the most important driver for cognitive deficits in both PDD and DLB. Using the genetic evidence, DLB represents a composite phenotype with respect to pathogenic processes in PD and AD. APOE ε4 is a susceptibility locus for general synucleinopathy and subsequent dementia regardless of AD pathology. This suggests that specific genetic factors contribute to deteriorating cognitive function and widespread aberrant protein accumulation in AD and Lewy bodies dementia. Potential genetic differences across loci found in both DLB and PD may imply disparate regional involvement of the synucleinopathic process in the former. Missense mutations in the synuclein gene and presenilin 1 and 2 underscore the importance of delineating nosological entities within the Lewy body disease spectrum, considering concurrent biomarkers, imaging, and genetic data [62]. Multimodal methodologies are promising for adopting a pharmacogenomics approach under the umbrella of personalized medicine for patients on the Lewy body disease spectrum.

A prospective cohort study included 78 PDD and 62 DLB patients who had diagnostic follow-ups for ≥3 years post-baseline. The clinic-demographic characteristics were used as predictors in addition to different neuropsychological tests (mini-mental state examination, PD cognitive rating scale, brief visuospatial memory test, symbol digit modalities test, Wechsler adult intelligence scale, trail making A and B) [63]. Logistic regression, K-nearest neighbors, support vector machine, naïve Bayes classifier, and ensemble model had a high prediction sensitivity, specificity, and accuracy of PDD or DLB diagnoses used non-invasively and easily in the clinic provided neuropsychological tests [63]. This could provide some help for the selection of suitable cognitive batteries in longitudinal follow-up studies and even allow a potential offer of more personalized therapeutic options or new elements to inform decision-making during clinical practice.

A 3D CNN method was developed, and external validation was performed to predict the final clinical diagnosis of AD, DLB, and MCI due to AD or CN with FDG-PET [64]. Further work will involve incorporating the proposed algorithm into clinical workflow as a decision support tool and including additional rationales to model outcomes to enhance explanations while improving transparency and trust. A machine learning algorithm mined from EEG could distinguish between DLB and AD [65]. This might be a novel biomarker for DLB. With real-world testing, a visually interpreted machine learning-generated scale for dopamine transporter function by single-photon emission computed tomography showed utility in distinguishing PD from CN and DLB from AD and PD from AD [66]. For improving diagnostic accuracy, further studies need to generate models with multi-center data pool.

Neurodegenerative diseases are largely focused on neuronal cell dysfunctions and their selective vulnerability; in most cases, they lack a global analysis regarding other contributing cellular subtypes. A systematic study unified spatial gene expression, structural MRI, and cell deconvolution to characterize large-scale spatial associations between canonical cell types and brain tissue loss across cortical and subcortical grey matter areas in 13 neurodegenerative diseases—early-onset and late-onset AD, PD, DLB, amyotrophic lateral sclerosis, mutations in presenilin 1, clinical (behavioral FTD, nonfluent variant primary progressive aphasia [PPA], semantic variant PPA) and pathological subtypes of frontotemporal lobar degeneration [67]. The spatially distributed microglia and astrocytes were intimately correlated with general tissue loss apparent in neurodegenerative diseases. Cells and disorders identify principal axes of spatial vulnerabilities, as cells map onto both different and shared clinical presentations. The whole-brain maps of cellular abundances open new avenues to disentangle the associations between imaging phenotypes and healthy reference levels in other neurological diseases [67]. In the future, artificial intelligence approaches should be incorporated into systematic analyses to facilitate differential diagnosis and improve our understanding of the underlying biology for targeted treatments, with a view to implementing them in clinical practice.

## 8. Challenges and Potential Solutions of Using Artificial Intelligence Approaches in the Clinic

Artificial intelligence can offer solutions for drug discovery and development of neurodegenerative diseases and clinical trial design through fostering cross-disciplinary, open science collaborations to evaluate existing (evidence) gaps and challenges [68]. Broad application of artificial intelligence can facilitate decision-making for drug discovery among academia, the pharmaceutical industry, and healthcare systems. Artificial intelligence can significantly expedite neurodegenerative disease drug development at various life cycles, including the choice, design, and synthesizing of molecules; the prediction of side-effects and drug interactions and contraindications; clinical and biological factors guiding the sample size considerations; data analyses of clinical trials; and the translation of trial efficacy to real word effectiveness by exposing individuals mostly likely to benefit from the interaction. Artificial intelligence may propel drug development and personalized medicine for individuals affected by neurodegenerative diseases [68]. The discovery of biomarkers that are accurate and financially viable in clinical settings will enable efficient screening of patients at risk of developing neurodegenerative diseases and effective tracking for better understanding of disease progression and accordingly stratify patients for treatments in the pipeline.

Since patients first see primary care physicians, these physicians are commissioned with the arduous task of diagnosing dementia and then delineating the exact cause of dementia (e.g., AD, DLB, and orphan diseases such as FTD) and commencing evidence-based treatment. The implementation of artificial intelligence approaches coupled with clinician perspectives will likely enable an effective dementia ecosystem to be established to prevent cycles of misdiagnosis through ensuring timely diagnosis and management, ultimately improving patient outcomes. Harnessing artificial intelligence platforms will help match diseases with potential treatments, a prime example of using artificial intelligence to assist drug repurposing or repositioning. Artificial intelligence algorithms can in the future be used to replace manual quantifications or traditional stereological approaches, methods prone to human user bias. It may also be used to identify phenotypic variations in pro- and anti-inflammatory glial cells in mammalian species in a precise manner [69].

The translation of artificial intelligence to the clinic is plagued with technical and disease-related challenges and institutional challenges (Table 3). A key challenge with the use of artificial intelligence in clinical practice will be generalizability. Overfitting is a common technical issue responsible for poor generalizability. Overfitting refers to a phenomenon in which the artificial intelligence model becomes overtly adjusted to the training data and learns not only the underlying patterns but also noise or small fluctuations. This is not an ideal situation, as there may be some information that performs well on the training data but fails to generalize to new patient-level data. This presents a problem when transitioning from research settings to real-world settings, where there might be a higher abundance of ethnically diverse populations with different demographics. Generally, an overrepresentation of Caucasians compared with other ethnicities (e.g., Black, Latino, and Asian) is observed in clinical trial settings. This is despite the likelihood of a 40% increase in AD among Latino/Black in the United States by 2030 [9,70]. One solution for circumventing the overfitting issue is to collect additional training data from ethnically diverse populations, but there is data scarcity in the field of dementia. ADNI4 is a key project that will enroll 50–66% of unrepresented populations with novel biofluids and technologies and may address the issue of generalizability with artificial intelligence [71]. Other initiatives, including UK biobank, AIBL, PPMI, and various registries, will be required to validate the ADNI findings.

Since the data pertaining to patients with dementia are highly enriched and multifactorial in nature, artificial intelligence developers are tempted to formulate complex models with multiple parameters, which may render the models susceptible to overfitting. This may result in the detection of small variations in neuroimaging, cognitive assessments, genetic tests, and other modalities with unknown clinical significance and may be artifactual. This implies that the predictive ability of the existing model in neuroimaging, for instance, will not translate to images acquired from other scanners or different clinical environments. In addition, it has been widely reported by different studies that patients with AD have co-morbidities, including the presence of α-synuclein pathology in up to 50% of cases [72,73]. It is not clear how heterogeneity in pathologic changes in AD may impact the training datasets and ultimately the functionality of the algorithms. Since no reliable gold standard exists for diagnosis, further work is required to establish the reliability of algorithm training before its implementation in the clinic.

While physicians believe that artificial intelligence can shape present and future medicine, as confirmed by most survey respondents (physicians working in hospitals) agreeing that artificial intelligence will have an appreciable impact within this decade, there are several concerns that need to be justified. The most expected benefit from the integration of AI was workflow improvements among radiologists, such as shorter reading time (~92%) or higher diagnostic accuracy with reduced error rate without missing positive studies (~73%). The major problems using artificial intelligence software were making wrong decisions owing to machine errors (~55%) and not having trust in judgement based on artificial intelligence approaches (~48%) [74].

Specialized training is required for dementia physicians and radiologists to understand how they should interpret artificial intelligence-driven results. This leads often to concerns or fears about how steep the learning curve will be for this innovation and if professionals can learn these new skills in time. Moreover, some are concerned that as artificial intelligence advances, physicians will have fewer chances to learn traditional diagnostic skills by training in reading MRI or PET scans manually. An international European survey reported the most common concern raised by radiologists and radiology residents was the decrease in professional reputation of radiologists (~60%) and fewer learning opportunities (~26%) when questioned about the integration of artificial intelligence in clinical practice [75]. This concern also affects medical students, of whom ~59% had selected radiology as their first choice and experienced anxiety over the impact of artificial intelligence on radiology [76]. Academic radiologists and medical schools should offer education to medical students on how artificial intelligence can positively influence radiology and complement physician decisions, rather than be discouraged by artificial intelligence [77].

Most artificial intelligence algorithms, particularly DL models that operate as “black boxes”, may make accurate predictions but provide little or no explanation of how those decisions are reached to the physician. Given the intricate nature of patient symptoms and imaging in dementia care, physicians need transparency over artificial intelligence decision-making to trust it being used effectively clinically. The need for transparency in artificial intelligence models—which does not bode well for how reliable they are. When physicians trust that a procedure has more than mechanical value, less explanation is necessary, and the acceptance of artificial intelligence system-based decisions will be easier even when guided by unsupervised DL procedures applied for image or feature recognition. This clearance is a significant step to ensure that artificial intelligence systems work smoothly with established clinical workflows. Dementia doctors, for example, are concerned that using artificial intelligence in radiology may raise the level of complexity to a diagnosis by introducing quality enhancement steps like data validation or re-inspection of results not agreed with clinical observations. For instance, if artificial intelligence generates a lot of false alarms or recommendations that necessitate deeper investigation, then it might increase workload. The physician may face the challenge of balancing any artificial intelligence suggestions with their expert knowledge, for fear of prolonging an arduous diagnostic process of dementia [74].

The most concerning ethical issues with artificial intelligence revolve around a patient’s confidentiality, disparities in access to diagnosis and treatments, accountability, and liability, essential to the clarity and confidence that any data create, or access will be protected as part of a private infrastructure. Data integrity and privacy need to be built on data transmission standards, such as the widely adopted Fast Healthcare Interoperability Resource for clinical data communication. Encryption approaches like homomorphic encryption and semantic hashing are indispensable to safeguarding the sensitive patient information. Federated learning is a possible solution that shields patient-level data by implementing common lessons learned across multiple local sites without requiring a raw, individualized original data transfer. But as federated learning is usually structured, a central server will be aggregating and updating the model parameters, which, in the presence of adversaries, could lead to information leaks. Swarm learning is a new paradigm introduced quite recently that integrates edge computing and blockchain’s P2P (peer-to-peer) networking-based properties, wherein it coordinates in an ad-hoc manner and there are no central coordinators. When it updates the model parameter, a site will push its local model parameters to all other parties through this mechanism by starting what is essentially a “transaction” via smart contract. Decentralized, large-scale multi-institutional clinical trials are facilitated by swarm learning, which ensures compliance with patient data collection and analysis developments with more security [68,78,79,80,81].

It is likely that artificial intelligence approaches become preferentially available to well-funded hospitals, leading to disparate access to diagnosis and treatment for under-resourced hospitals/community centers. This may lead to local, regional, and international inequalities in care for patients with dementia. Screening programs find many people with suspected disease who require additional follow-up tests to establish a diagnosis. When the early detection program is implemented, episodic patient pathways must be anticipated within the health service and resources allocated accordingly. Many health systems, however, are underfunding mental health services with a limited capacity to serve the demand. Although it is hoped that this earlier detection has long-term financial benefits to health care, current systems are ill equipped for the tsunami of patients funneled into memory assessment services following screening. Therefore, to be effective and ethical, such early detection programs are dependent on prior investment in dementia diagnostic and support services. This is problematic in artificial intelligence approaches, particularly in the field of digital biomarker development, as some measurements may be minimally expensive to capture but still bear costs through these cascading layers across health.

Other thoughtful aspects relate to the psychological support when informing about personal risk of dementia. Dementia today is a progressive illness and has implications for future health and mental capacity. Hitherto, evidence regarding the risk of receiving predictive information associated with dementia for asymptomatic individuals (i.e., secondary disclosure or genetic testing) has been in combination with pre- and post-test counselling and professional support. After all, this type of essential support may only be needed temporarily, and at present there are few trials that might yield information on the benefit vs. harm implications for the unsupported return of results. Additionally, once results have been obtained, interviewees and the broader patient populations may need (and are entitled to) support monitoring and follow-up as part of the service, which would require accounting for in cost projections when planning a systemic impact via digital biomarkers.

While ethical grounds are favorable for detecting early AD with the recent availability of anti-Aβ treatments across the United States, they are less favorable in Europe with the recent rejection of anti-Aβ treatments. The next question is whether there are sufficient ethical grounds for the detection of asymptomatic individuals. The ethical grounds supporting early detection are that it may stimulate the individual to adopt a healthy lifestyle, exercise, and plan for the future. From an academic and clinical standpoint, better understanding of how at-risk individuals in the absence of symptoms longitudinally progress in AD pathogenesis is informative. The ethical grounds against the detection of asymptomatic individuals are the absence of disease-modifying therapies, more so in Europe; while in the United States, anti-Aβ treatments are approved, but their effectivity in the real world needs to be proven. Moreover, the real-world monitoring of ARIA in patients taking anti-Aβ is essential, and the risk of ARIA-E and ARIA-H is not trivial and can be severe/fatal in rare cases. Physicians should ensure patients make informed choices prior to taking anti-Aβ treatments after carefully evaluating the benefits of anti-Aβ treatments vs. the risks of particularly developing ARIA. Though the predictive artificial intelligence systems tend to have good accuracy, there is still no social consensus regarding how much predictive power should be reclaimed. Testing is clinically inappropriate in the absence of regulatory approval due to incompletely validated techniques that currently produce false negatives and positives. There is no consensus regarding the threshold for predictive utility. The amyloid cascade hypothesis remains unproven. Patients may misinterpret prognostic information, creating therapeutic misconception. However, false-negative diagnoses could be misleading and cause a patient to feel falsely reassured; they might then avoid treatment or clinical trials. False-positive diagnostic tests could result in over-diagnosis, unnecessary treatment, mistaken entry onto clinical trials, and injurious invasive biomarker testing. The risks of screening asymptomatic individuals are stigmatization and discrimination at work or by insurance companies, confiscation of driving licenses, employment discrimination, and social seclusion. There is a robust case for ensuring that there is and remains such a thing as the right not to know. Reasons for refusing to undergo biomarker status assessment are fear of AD and lack of disease-modifying treatment, especially in European countries, and a cautious approach is adopted despite the availability of disease-modifying treatments in the United States. While up to 81% of physicians in German hospitals agree on a patient’s refusal as an option, almost all (75%) practice disclosure not only with core information but with other results routinely. In addition, 10% communicate results only upon request, and the same proportion communicates them but together with psychological counselling [82]. Conversely, an ethos of non-maleficence is articulated pertaining to a culture of reluctance around disclosing dementia diagnosis [83]. Such behavior might be to play far away, being paternalistic, but it could also have negative side effects dealing with a well-intentioned physician not revealing information. The principle of autonomy requires that study participants have the ethical right to a full disclosure upon request. There is an unmet need to provide structured training to physicians to enable counselling of patients regarding artificial intelligence and machine learning systems. Standardized guidelines of good practice on how to disclose information to patients while adhering to data protection safeguarding methods are vital. Taken together, ethically, predictive testing must be governed by ethical principles designed to protect the interests of both individuals and society. Within a clinical case, these principles and their claims can be utilized to help deliberate on the different concerns by balancing them against each other. The most essential principle at the individual level is autonomy, which can be ensured by asking whether a subject has the desire to undergo predictive testing. In summary, predictive testing should only be available to asymptomatic individuals as part of a research use case (and under the purview and competency of a medical geneticist) upon request [79]. While currently a risk to the individual, governments must ask: what is the continuing prevalence of disease so they can prepare for future social services? The challenge is a tricky clinical–societal tradeoff: the test yields clinically and socially valuable data, despite a lack of disease-modifying treatments. Thus, it is unclear if the use of artificial intelligence and machine learning algorithms to detect individual at-risk subjects in population-level screenings would be ethically justified.

**Table 3:** Barriers towards clinical implementation of artificial intelligence approaches to neuroimaging in neurodegenerative diseases and the desired future behaviors [84].

Barriers	Current State	Desired Future Behaviors
Technical	Challenging to generalize artificial intelligence approaches across different scanner types across hospitals, different patient subgroups, and characteristics (e.g., age or sex)	Larger datasets, methods that prevent overfitting, domain switching, and harmonization
	Ready-to-use software products and current systems may struggle to handle high computational requirements that are expensive	Investment in software engineering and user experience, corporate partnerships, cloud-based solutions, and institutional investment in servers
	Incomplete electronic health records, use of different formats—unstructured and structured, rendering it difficult to train artificial intelligence models	Meticulous and accurate record keeping, translating clinician notes, and development of methods and/or imputation approaches to handle incomplete or mislabeled data
Disease-related	Lack of biomarkers, insufficient neuroimaging modalities, and lack of disease differentiation	Multimodal techniques to discover biomarkers, which include inclusion of neuroimaging tools into clinical workflows and diagnostic algorithms to differentiate between diseases
Institutional	Clinicians are apprehensive of using new methods due to the technical skills required and time limitations; databases (electronic health records) are often incomplete and mislabeled; lack of capabilities for surveilling of effectiveness of artificial intelligence models and patient confidentiality is a concern	Specialized programs to partner artificial intelligence experts with clinicians to prioritize usability; institutional investment in clinical databases (developing structured electronic health records); centralized monitoring and reporting systems to ensure the effectiveness of models and patient confidentiality

If artificial intelligence is used in the diagnosis of dementia, the issue of accountability between the physician, institution, or the artificial intelligence developer becomes complicated if the diagnosis is incorrect. A misdiagnosis in dementia is not trivial and may have a significant effect on a patient’s life and decision-making process.

Although artificial intelligence has the potential to improve diagnosis for dementia, such as in radiology, there remain legitimate concerns from clinicians regarding the validity of results produced by these models and workflow integration or ethical issues. Meeting these challenges and accelerating adoption in dementia care will hinge on artificial intelligence’s ability to enhance rather than replace clinical acumen. It is imperative that the artificial intelligence models are gradually scaled across hospitals with adequate training provided to healthcare professionals and local technicians. An ecosystem for data scientists, software engineers, and healthcare professionals should be established to enable effective and efficient use of artificial intelligence software [84]. To enhance adherence and acceptance of artificial intelligence software by the physicians, artificial intelligence developers should ensure good communication practices with physicians to de-jargonize the language of artificial intelligence to maximize physician acceptance. A strategy that can be favorably deployed at symposiums or seminars is for artificial intelligence developers to deal with feedback exchanges from physicians in real time to assist with co-development/co-validation pertinent to the clinic.

## 9. Conclusions

Artificial intelligence methods offer the unprecedented opportunity to classify, diagnose, and monitor neurodegenerative disease progression in a timely fashion. Further development and refinement of these models will be required to overcome challenges including generalization and data deficiency, absence of in vivo gold standard, patient and healthcare professional attitudes to the changing landscape, apprehensions over patient confidentiality, and safety. The implementation of artificial intelligence methods in clinical practice can potentially transform the diagnostic and treatment landscape and improve patient health and outcomes.

Multimodal machine learning infrastructure can give an entire toolset to precision medicine drug discovery in AD that may encompass data-driven target identification from all of the “-omes” disease subphenotypings, speeding up clinical trial testing for disease-modifying treatments; identifying molecular mechanisms and predicting underlying effectiveness through preclinical assays (such as pharmacodynamics, brain penetration by CNS cell membrane permeability, etc.) and ADMET properties, with emphasis on absorption, distribution, metabolism, excretion, and toxicity profiling; automated drug-like liabilities scoring along with patient specific selection criteria, followed by candidate molecule generation using a combination of artificial intelligence/ML techniques from deep generative models, reinforcement learning, graph networks, etc.; and fractionated real world observations aimed at reviewing efficacy and quality assessment against performance standards to ensure timely enrichment for further lead optimization and providing mechanistic insights into biology-to-clinic resistance mechanism may facilitate personalizing early sequential evaluation steps.

A real-world clinical adaptation of artificial intelligence-guided tool for prediction of early dementia, applied directly to memory clinic patient data has the potential to reduce misdiagnosis in people with mild symptoms at initial presentation; standardize diagnostic approach across different sites which should also help minimize healthcare disparities between centers and allow invasive/difficult investigations (lumbar punctures, etc.) only on patients where this is clearly warranted; focus services more appropriately towards those who need most assistance and are most likely to respond well due continuation/supportive therapy interventions when these modalities may have best effect. Moreover, assistive artificial intelligence approaches may be used to complement physicians and enhance the diagnostic performance of ARIA. The assistive software does not eliminate the core problem of radiologists in reading brain MRIs. Better detection of ARIA and safety management for patients on the anti-Aβ antibody therapies will be possible only when more sensitive, less subjective, and more reliable methods to identify it up front become available with wider awareness in our radiology/clinical teams and regarding appropriate use of theatrical assistive software tools.

Future integration of generative and explainable artificial intelligence will ensure timely and improved diagnostic accuracy through analyses of numerous and large datasets. It will also facilitate precision and personalized medicine, as well as accelerate drug discovery and clinical trial designs in a cost- and time-effective manner. The synergistic potential of generative and explainable artificial intelligence may achieve synthetic data transparency, decision justifications solving physician trust issues, and custom models catering to specific dementia intricacies.

## Figures and Tables

**Table 1:** Select artificial intelligence approaches used in Alzheimer’s disease.

Method	Result
Machine learning [23] fMRI N = 119; 44 AD, 30 MCI, 45 CN	Classification accuracies of 82.0% (AD vs. CN), 81.3% (MCI vs. CN), and 81.08% (AD vs. MCI)
Deep learning [24] MRI N = 968; 280 AD, 162 MCI-c, 251 MCI-nc, 275 CN	Classification accuracies of 97.4% (AD vs. CN), 87.8% (MCI-c vs. CN), and 78.8% (MCI-c vs. MCI-nc)
Machine learning [25] MRI N = 339; 143 FTD, 50 AD, 146 CN	Classification accuracies of 86.1% (CN vs. FTD + AD), 90.8% (FTD vs. AD), 86.9% (bvFTD vs. PPA), and 92.1% (svPPA vs. nfvPPA)
Machine learning [26] rs-fMRI, DTI N = 60; 33 AD, 27 VD	84.0% classification accuracy and a correct prediction rate of 77.3%
Deep learning [27] MRI, FDG-PET N = 1242; 360 sCN, 409 MCI-nc, 18 pCN, 217 MCI-c, 238 sAD	84.4% accuracy in identifying MCI-c 3 years prior to conversion to AD; 94.2% sensitivity in classifying clinically probable AD; 86.3% specificity in classifying CN
Deep learning [28] fMRI N = 138; 25 CN, 13 MCI, 25 EMCI, 25 LMCI, 25 SMC, 25 AD	Classification accuracy of nearly 100% on EMCI vs. LMCI, EMCI vs. AD, LMCI vs. AD, and MCI vs. EMCI; 98.7% (CN vs. EMCI), 92.2% (CN vs. LMCI), 80.8% (AD vs. CN)
Deep learning [29] FDG-PET N = 496; 190 AD, 134 FTD, 172 CN	Areas under the ROC curves were 93.3% for AD, 95.3% for FTD, and 99.9% for CN

AD, Alzheimer’s disease; bvFTD, behavioral variant FTD; CN, cognitively normal; EMCI, early MCI; FDG, fluorodeoxyglucose-positron emission tomography; fMRI; functional magnetic resonance imaging; FTD, Frontotemporal dementia; LMCI, late MCI; MCI, mild cognitive impairment; MCI-c, MCI-converters; MCI-nc, MCI non-converters; MRI, magnetic resonance imaging; nfvPPA, non-fluent/agrammatic variant PPA; pCN, progressive CN; PPA, primary progressive aphasia; ROC, receiver operating curve; rs-fMRI, resting state-fMRI; sCN, stable CN; svPPA, semantic variant PPA; VD, vascular dementia.

**Table 2:** Gene-SGAN identifies four subtypes of brain changes related to Alzheimer’s disease (A1, A2, A3, and A4), which show distinct imaging, genetic, and clinical characteristics [37].

	Imaging Voxel-Based Morphometry	Genetic ^a^	Clinical
A1	Preserved regional brain volumes	Lowest frequency of rs429358, rs9469112, rs9271192	Lowest levels of cognitive impairment and β-amyloid/tau deposition, indicating a resilient subtype
A2	Focal medial temporal lobe atrophy, particularly hippocampus and anterior-medial temporal cortex	Highest frequency of rs429358 High frequency of rs9469112 and rs9271192	High cerebrospinal fluid tau, suggesting a subtype driven by limbic-predominant, likely rapidly progressive neuropathology
A3	Widespread brain atrophy and highest white matter hyperintensity volumes	High frequency of rs9469112 and rs9271192	Most abnormal cerebrospinal fluid β-amyloid, the worst cognitive performances, suggestive of typical Alzheimer’s disease pathology and vascular co-pathology
A4	Dominant cortical atrophy with relative sparing of the medial temporal lobe	Highest frequency of rs7920721	Subtype suggestive of a mixture of participants with a cortical (atypical) presentation of Alzheimer’s disease and those with other neural degenerative processes (e.g., advanced brain ageing); significantly low age range indicative of early-onset Alzheimer’s disease

^a^ rs429358 was mapped to the apolipoprotein E gene; rs9469112 and rs9271192 were mapped to human leukocyte antigen region that was involved in immune response modulation; rs7920721 was exclusively associated with Alzheimer’s disease among participants who do not carry apolipoprotein E ε4 isoform.

## Data Availability

No new data were created or analyzed in this study. Data sharing is not applicable to this article.
